# Predictors of Diet Quality as Measured by Malaysian Healthy Eating Index among Aboriginal Women (Mah Meri) in Malaysia

**DOI:** 10.3390/nu11010135

**Published:** 2019-01-10

**Authors:** Su Pei Chong, Geeta Appannah, Norhasmah Sulaiman

**Affiliations:** Department of Nutrition and Dietetics, Faculty of Medicine and Health Sciences, Universiti Putra Malaysia, Serdang, Selangor 43400, Malaysia; sharenchongsupei@gmail.com (S.P.C.); geeta@upm.edu.my (G.A.)

**Keywords:** diet quality, healthy eating index, socioeconomic status, nutrition knowledge, Malaysian aborigines

## Abstract

Socioeconomic status and nutrition knowledge are the determining factors of food choices. However, their relationship with diet quality is ambiguous among aboriginal women in Malaysia. Henceforth, the objective of this study was to examine diet quality and its predictors among the aboriginal women from the Mah Meri ethnic group in Malaysia. Data on socioeconomic characteristics, nutrition knowledge, and 24-h dietary recalls were obtained through face-to-face interviews with the respondents. Household food insecurity was assessed using Radimer/Cornell Hunger and Food Insecurity Instrument. The Malaysian Healthy Eating Index (HEI) was used to measure the diet quality of this population. The overall diet quality of the respondents was poor, with a mean Malaysian HEI score of 45.3%. Household income (*r* = 0.242, *p* < 0.001) and nutrition knowledge (*r* = 0.150, *p* < 0.05) were positively correlated with diet quality. More importantly, the predictors of diet quality were marital status (*β* = 0.181, *p* < 0.01), household income (*β* = 0.237, *p* < 0.001), food security status (*β* = −0.151, *p* < 0.01), and fat intake (*β* = −0.438, *p* < 0.001). Women being married and those with higher household income was associated with a better diet quality among Malaysian aborigines.

## 1. Introduction

Malaysia is facing a rise of diet-related non-communicable diseases (NCDs) [1], imposing socioeconomic burdens, especially among lower income households [2]. An updated meta-analysis revealed that good diet quality was associated with significantly decreased risks of NCDs and all-cause mortality [3]. It was well documented that NCDs could be prevented by dietary components, such as lowering the consumption of refined grains and increasing the intake of whole grains, fruits, vegetables, and legumes, which were associated with decreased risk of obesity and type 2 diabetes [4]. Reduced sodium intake was associated with decreased risk of hypertension and cardiovascular diseases (CVD) [5]. The accumulated scientific evidence clearly showed that the intake of vegetables and fruits had protective effects against CVD risks, however, Alissa and Ferns suggested that vegetables and fruits should be taken as part of a balanced diet, because a set of nutrients may interact with genetic factors synergistically to reduce CVD risks [6].

Conventionally, one single nutrient or food group was used in studies to examine the associations between diet and health. However, realizing that an individual’s complete diet is comprised of a variety of foods, a new approach of studying dietary patterns, and consequently diet quality, had emerged to better capture the interactions and synergistic effects of foods and nutrients on health [7]. The Healthy Eating Index (HEI) is one of the indexes of overall diet quality based on both nutrients and food groups, unlike indexes that are based on either nutrients or food groups only, for example Nutrient Adequacy Ratio and Diet Diversity Score, respectively. The Malaysian HEI is one of the many existing indicators to measure the overall diet quality of Malaysian adults based on the degree of compliance to dietary recommendations in the Malaysian Dietary Guidelines (MDG) [8].

Several studies in America and Canada have found that food insecurity was associated with diet quality as measured by Healthy Eating Index [7,9,10]. Multiple demographic and socioeconomic characteristics, including age, sex, marital status, education, occupation, and income, were also shown to be associated with diet quality [11]. Various epidemiological studies depicted that the quality of diet followed a socioeconomic gradient, suggesting that the social disparities in diet quality may be explained by food prices and diet cost [12]. Individuals with lower socioeconomic positions were found to be associated with lower diet quality, as characterized by low consumption of nutrient-rich foods and high intake of energy-dense foods [13]. Nutrient-rich foods are defined as foods with a high content of vitamins and minerals, such as vegetables, fruit, lean meat, and fish. Conversely, energy-dense foods are food high in calories, such as refined grains and fats [12].

In Malaysia, the aboriginal people are called *Orang Asli*. Based on our current knowledge, this was the first attempt to study the diet quality of *Orang Asli* women using the Malaysian HEI. This study also aimed to determine the relationship between socioeconomic characteristics, nutrition knowledge, food security status, and diet quality among this vulnerable group.

## 2. Materials and Methods

### 2.1. Study Population and Design

This cross-sectional study was carried out among *Orang Asli* (Mah Meri) women aged between 19 to 59 years old within the district of Kuala Langat in the state of Selangor. Using a cluster sampling method, two sub-districts, named Jugra and Batu, were randomly selected in this study. All households within the villages under these two sub-districts were recruited in the study. Each household was represented by a woman. The total sample size required for this study was 216 respondents, which was inclusive of 20% expected non-response rate. The inclusion criteria were *Orang Asli* women from the Mah Meri tribe, and aged between 19 to 59 years, while the exclusion criteria were pregnant women, lactating mothers, post-partum women, vegetarians, and those suffering from acute or chronic illnesses, as well as individuals who had a change in food habits in the past six months to reduce weight. A total of 262 households that fulfilled the inclusion criteria were eligible for participation. However, only 222 (84.7%) households agreed to participate in this study. All the participants in the study signed the written informed consent form.

Prior to data collection, ethical approval for research was obtained from the Ethics Committee for Research involving Human Subjects of Universiti Putra Malaysia, IRB No. UPM/TNCPI/RMC/1.4.18.1(JKEUPM)/F2. Permission to carry out the study among Mah Meri households in Kuala Langat District, Selangor was approved by the Department of *Orang Asli* Development (JAKOA). Visits to Mah Meri households were arranged by the JAKOA officers, which had the full cooperation from the chairman and members of Village Security and Development Committee (JKKK). The village headmen (*Tok Batin*) were sought for guidance each time before entering the village. Data were collected between August, 2015 and January, 2016.

### 2.2. Socioeconomic Characteristics and Nutrition Knowledge

All respondents involved in this study were interviewed face-to-face regarding their socio-demographic information, such as their age, marital status, education level, employment status, and household income, for the purpose of understanding their background. Nutrition knowledge was assessed using a validated questionnaire developed by the Malaysian Technical Working Group on Research (TWG-R) [14]. This questionnaire contained 20 items that were comprised of five components, namely nutrient function, energy of food, nutrient insufficiency, food selection, and supply of nutrients. Each multiple choice question of nutrition knowledge had five answers. Scores on the knowledge were calculated by giving one mark to each correct answer. However, no marks were given for incorrect or void responses. The minimum achievable score was zero while the maximum score was 20, and these scores were then converted into percentages. The total score was 100 percent, in which less than 51% indicated poor knowledge, 51 to 74% indicated knowledge at a moderate level, and more than 74% indicated good knowledge [14]. The Cronbach’s alpha of the instrument used in this study was 0.88, which indicated high reliability.

### 2.3. Food Security Status

The Radimer/Cornell Hunger and Food Insecurity Instrument was used to examine the severity level of food insecurity in a household. This questionnaire was developed by Radimer et al. (1990) [15] and further study was conducted to establish the validity and reliability of the instrument [16]. The food insecurity construct consists of four components, which are quantity, quality, psychology, and social. Food insecurity usually started at the household level (food anxiety about the sufficiency of food supply), followed by adult food insecurity (food quantity and quality compromised), and child hunger (decrease in food quantity). There were 10 items, and each item was given three choices, which were “never happen”, “sometimes happen”, or “always happen”. The first level was food security: negative answer “never happen” to all items. The second level was household food insecurity: positive answer “sometimes or always happen” to at least one of items 1 to 4. The third level was individual food insecurity: positive answer to at least one of items 5 to 8. The most severe level was child hunger: positive answer to items 9 and 10 [15,16]. In this study, the Cronbach’s alpha of the instrument was 0.85, indicating high reliability.

### 2.4. Dietary Intake Assessment

A 2-day 24-h dietary recall method, including one weekday and one weekend, was used in this study to determine food intakes of respondents. The dietary recall method was carried out by face-to-face interview. Respondents were asked to recall all foods and drinks consumed in the last 24 h. To obtain a more accurate estimate of the serving sizes during the interview, household measures such as teaspoons, tablespoons, cups, bowls, and glass were used, along with the use of the Food Atlas: Size Portion and Exchange [17], as well as the Malaysian Food Album [18]. Meal time, type of meal, quantity taken, methods of cooking, and recipes of some of the dishes were also recorded.

All the data obtained were analyzed using the Nutritionist Pro^®^ Software Version 4.0 (Axxya Systems, Stafford, TX, USA) and were compared with the Recommended Nutrient Intake (RNI) for Malaysia [19]. If the food consumed were not listed in the Nutritionist Pro^®^, then information about the food nutrients were obtained by referring to the food listed in the Nutrient Composition of Malaysian Food [20] and Association of Southeast Asian Nations (ASEAN) Food Composition Database [21]. As shown in Supplementary Table S1, the serving size for each food group was determined based on the Food Atlas: Size Portion and Exchange [17], the Malaysian Dietary Guidelines [22], and the Guidelines for Serving of Healthy Meals during Meeting [23]. For the cooked dishes and meals, some recipes were created in the Nutritionist Pro^®^ program to complement several cooking styles of the *Orang Asli*, which were different from the cooking styles of the regular cooked dishes in the Malaysian food composition data. A total of 76 recipes of local delicacies were created, such as “wild boar curry”, “banana blossom cooked in coconut milk”, and “mackerel fish cooked with fermented durian”.

### 2.5. Dietary Misreporting

The ratio of reported energy intake to basal metabolic rate (EI: BMR) was calculated to determine the under- and over-reporters of energy intake. The reported EI was calculated based on the average value of energy intakes estimated from the two days with 24 h diet recall. The BMR for each respondent was calculated based on the prediction equation for healthy women aged between 18 to 60 years in Malaysia by Ismail et al. (1998) [24]. Using the Goldberg cut-off, as was restated by Black (2000) [25], the factor (S) took into account the within-respondent variations in EI (CVwEI), number of days of diet assessment (d), within-respondent variations in BMR (CVwB), and between-respondent variations in physical activity level (PAL) (CVtP). The revised factor by Black (2000) should be applied according to 2-day diet assessment: CVwEI = 23%; CVwB = 8.5%; CVtP = 15%.

$$S=\sqrt{\frac{{\mathrm{CVwEI}}^{2}}{2}+{\mathrm{CVwEI}}^{2}+{\mathrm{CVtP}}^{2}}$$

$$S=\sqrt{\frac{23^{2}}{2}+8.5^{2}+15^{2}}=23.7$$

The recommended energy requirements for adults by the World Health Organization (WHO) was based on PAL 1.55, thus this value was included in the equation. The values of SD min of -2 for the 95% lower confidence limit and SD max of +2 for the 95% upper confidence limit, with n = 1, were used to identify misreporting at the individual level. So, the lower cut-off for this study was

$$\mathrm{EI}:\mathrm{BMR}>1.55\times \exp\;\left[-2\times \frac{\left(23.7/100\right)}{\sqrt{1}}\right]$$

EI:BMR < 2.49

and upper cut-off for this study was

$$\mathrm{EI}:\mathrm{BMR}<1.55\times \exp\;\left[2\times \frac{\left(23.7/100\right)}{\sqrt{1}}\right]$$

EI:BMR < 2.49

In this study, respondents with calculated EI:BMR in the interval of 0.96 to 2.49 were classified as acceptable reporters. Respondents with EI:BMR < 0.96 were categorized as under reporters, whereas respondents with EI:BMR of >2.49 were over reporters.

### 2.6. Malaysian Healthy Eating Index Scoring

The Malaysian HEI was developed by Lee et al. (2011) [8] to assess the overall diet quality of the respondents in Malaysia, and it was validated by Goh and Norimah (2012) [26]. The instrument consisted of nine components, which was made up of seven food groups and two nutrient groups, as shown in Table 1. The scoring of these components was calculated based on the recommended serving size and nutrient intake in the MDG [22]. The score of each food group was calculated using the formula: (actual serving consumed based on respondent’s diet recall/recommended serving size based on MDG) × 10. For example, if a moderately active woman consumed 4 servings of grains and cereals, then the score was calculated as 4 servings divided by 6 servings, as per the recommendation in the MDG (refer to Supplementary Table S2), and multiplied by 10. The score of each nutrient ranged from 0 to 10, which was calculated proportionately for the in-between whole number responses, as shown in Table 2. The total score of HEI was obtained by summing up the score of each component. The composite score in percentage was calculated using the formula: (total score obtained from 9 components/maximum score of 90) × 100%. Therefore, the total score was 100%, in which less than 51% indicated poor diet, 51 to 80% indicated diet requiring improvement, and more than 80% indicated good diet [8]. The scoring of the instrument was published in the authors’ previous paper [27].

### 2.7. Data Analysis

The data obtained were analyzed using IBM SPSS Version 22.0 (International Business Machines Corporation, Armonk, NY, USA). Descriptive statistics were used to present mean, standard deviation, median, interquartile range, frequency, and percentage of all variables. The Pearson correlation coefficient was used to determine the correlation between the total score and the component scores of the Malaysian HEI with the socioeconomic and nutrition knowledge data. However, the Spearman rank correlation coefficients test was used to analyze the data that was not normally distributed. Simple linear regression (SLR) was performed to determine the relationship between an independent variable (socioeconomic characteristics, nutrition knowledge, food security status, and adjusted macronutrients) with a dependent variable (diet quality). To identify factors contributing to the outcome variable (diet quality), multiple linear regression (MLR) analysis was performed. Those variables showing statistically significant relationships in the SLR analysis (refer to Supplementary Table S3) were included in the MLR model, except for the education variable and adjusted protein variable, due to a multicollinearity problem. Results from the SLR and MLR models were expressed as unstandardized coefficient B with a 95% confidence interval. The significance level was set at *p* < 0.05.

## 3. Results

### 3.1. Socioeconomic Characteristics, Nutrition Knowledge, and Food Security Status

Table 3 shows the socioeconomic characteristics, nutrition knowledge, and food security status of the respondents. There were 222 respondents involved in this study and half of them were between 30 to 50 years old. The majority of the respondents were married and the remaining were either single, divorced, or widowed. Almost half of the respondents completed primary schooling, one-third of them attended secondary school, and 15.8% never attended school. Around 37.8% were employed, whereas 62.2% were housewives. The mean monthly household income was United States Dollar (USD) 228.2 ± 215.9. Most of the respondents lived within hardcore poor households (≤USD 140.1) and 26.1% were considered poor, with a household income of ≤USD 224.6 [28]. Additionally, the majority of the respondents were categorized as having poor nutrition knowledge, 14.0% as having moderate knowledge, and only 3.1% had good nutrition knowledge. About 82.9% of the households experienced some form of food insecurity, which were household food insecurity, individual food insecurity, and child hunger.

### 3.2. Energy and Nutrient Intake by Age Group

The 2-day 24-h dietary recall method was used to determine the energy and nutrient intakes of the respondents (Table 4). The energy intake of the respondents was approximately 80%, which was sufficient for all age groups based on the Recommended Nutrient Intake (RNI), whereas protein intake exceeded the RNI. The mean intake of iron, vitamin A, and vitamin C was adequate for the age group of 51–59 years, while the younger age groups did not meet the RNI. Additionally, the mean intake of calcium, zinc, selenium, thiamine, riboflavin, and niacin was insufficient for all age groups, as the respondents’ consumption only met around 40 to 70% of the RNI. In general, older women achieved greater numbers of nutrient intake compared to their younger counterparts.

### 3.3. Household Income and Nutrition Knowledge Correlated with Components of Malaysian HEI

As per Table 5, the overall diet quality of the respondents was poor, with a mean Malaysian HEI score of 45.3 ± 7.5%. A majority of the respondents were not able to meet the recommended serving size of vegetables, fruits, poultry, meat and eggs, legumes, milk, and dairy products. The total fat had a mean score of 7.3 ± 4.0 and sodium had a mean score of 7.4 ± 2.7. Among the socio-demographic variables, only household income was positively correlated with the Malaysian HEI scores of grains and cereals, vegetables, fruits, meat, poultry and eggs, and legumes. Nutrition knowledge was also found to be positively correlated with Malaysian HEI, especially the intake of vegetables and fruits.

### 3.4. Predictors of Diet Quality

Table 6 depicts the predictors of diet quality using the multiple linear regression model. About 41.6% of the total variance in diet quality was accounted for by the respondents’ marital status, household income, food security status, and intake of adjusted fat (*R*^2^ = 0.416, *p* < 0.001). Women with characteristics of being single, divorced, or widowed, living in lower income households, experienced food insecurity, and having a higher intake of adjusted fat had poorer diet quality.

## 4. Discussion

In this study, only 30.6% of the *Orang Asli* completed secondary school, which was less than half the national average of 72.0% [29]. Around 37.8% of the Mah Meri women were working, with a majority of them engaging in fishing-related occupations and jobs related to cultural activities, such as dancing, weaving, and carving [30,31]. Among the Mah Meri households, 26.1% were considered to be poor and 36.8% were hardcore poor, which was far higher than the national poverty rate of 3.8% for the poor and 0.7% for the hardcore poor [32]. This study indicated that the diet quality of the Mah Meri women was poor (lower HEI score), specifically for vegetables, fruits, meat, poultry and eggs, legumes, and dairy product components. Factors such as marital status, household income, food security status, and fat intake influenced the diet quality of the Mah Meri women in this study.

The respondents in this study reported poor diet quality compared to their non-indigenous women counterparts (Malay, Chinese, and Indian) [26,33,34]. This condition could be explained by the majority of the respondents who were not able to achieve the recommended minimum servings of food groups in their diet, including vegetables, fruits, meat, poultry and eggs, legumes and milk. In this study, the respondents consumed less vegetables (1.1 serving) and fruits (0.1 serving) as compared to the average Malaysian women who consumed vegetables (1.5 serving) and fruits (1.4 serving) [35]. The low consumption of vegetables and fruits could be due to the limited variety of vegetables around the *Orang Asli* settlements, and most of the available fruits in the area were seasonal ones, such as rambutan and mangosteen. Vegetables and fruits were infrequently consumed, as they could not afford to buy local and imported vegetables and fruits at the market. They mostly consumed wild vegetables (tapioca shoots and swamp cabbage) and non-seasonal fruits, such as banana and papaya, which were either grown locally or gathered in the forest [36].

Furthermore, the respondents in this study consumed 0.6 serving of meat and 0.1 serving of legumes, which was considered to be less satisfactory based on the MDG. They rarely consumed chicken and other meats sold in the market due to the high prices. They also seldom consumed legumes and milk products due to the lack of availability, acceptability, and cultural practices that limited the choices of these food items in their diet [36,37]. Like any other Malaysian, the *Orang Asli* also liked to consume coffee, tea, and malted beverages added with sweetened condensed milk or evaporated milk during breakfast and teatime [38]. The low Malaysian HEI scores for the components of vegetables, fruits, meat, legumes, and milk products could be attributed to nutritional inadequacy, whereby a majority of the respondents did not meet the RNI for minerals (calcium, iron, zinc, and selenium) and vitamins (thiamine, riboflavin, and niacin). Older women obtained higher nutrient intakes compared to their younger counterparts, as they were not only equipped with better knowledge and understanding of nutritional benefits, but they also had more experience in preparing nutrient-rich meals [39].

Nutrition knowledge was found to be positively correlated with diet quality, especially the intake of vegetables and fruits, as well as the overall diet quality [40,41]. Within the *Orang Asli* culture, the women (wives) traditionally play the caretaker’s role, who prepare meals and look after the family [42]. The low education attainment among the *Orang Asli* women posed a serious limitation for acquiring nutrition knowledge [43]. Women with low nutrition knowledge may have inadequate awareness and understanding of nutrition information, probably due to their illiteracy and language barrier, as most of the food labels, as well as commercial and educational materials on healthy foods, were in English [14,44]. Women with lower education most probably had poor nutrition knowledge and low awareness and understanding of nutrition information. In addition to lower income, they were predisposed to select food which were cheaper, thus their diet quality might have been compromised [40,45].

Poor diet, as measured by the Malaysian HEI, was associated with women either being single, divorced, or widowed. This result corresponded with a study by Alkerwi et al. (2015) [11] that found Luxembourg women living alone (single, divorced, or widowed) were associated with poorer dietary diversity score. The possible explanation might be the lack of family support and limited financial resources, which restricted their access to a variety of food choices [11]. In addition, several other studies found that food insecure respondents from lower-income households were associated with poor diet quality, and this finding could be explained by food prices and diet cost [9,10,12]. Individuals from food insecure households made choices of food that best served their own utility, depending on their daily energy requirements, socio-economic status, as well as budget allocated for food [12,46]. The high food prices and the financial constraint faced by the food insecure households not only limited the food choices, but also caused an overall reduction in food intake, and ultimately, poor diet quality [34,47].

Furthermore, the increment of the Consumer Price Index of food and non-alcoholic beverages (4.6% from December 2014 to December 2015) was mainly applied to healthy food groups, such as vegetables, fruits, meat, poultry and eggs, as well as fish [48]. These healthy food groups were less consumed mainly due to the cost factor, as healthy food items were more costly than the less healthy options [49]. Therefore, due to budget constraints, consumers from low-income households could only afford less healthy food groups, which were usually energy dense and nutrient poor food items, such as refined grains and fats [50]. This is in agreement with the results in this present study, which showed that an increased fat intake was associated with poor diet quality.

This cross-sectional study could not determine the cause–effect relationship, and the findings from this study could not be generalized to the rest of the *Orang Asli* populations in Malaysia. The 2-day 24-h dietary recall method was used to estimate the dietary intake of respondents; thus, the data was not based on accurate dietary intake. Therefore, there was a likelihood of data misreporting due to the respondents either under- or over-reporting their dietary intake. This study showed that 31.5% of the respondents were under-reporters. The Malaysian HEI used to measure diet quality did not evaluate the excess levels of carbohydrate and protein intake. Nevertheless, to the best of our knowledge, this was the first study that attempted to assess diet quality using the Malaysian HEI among Orang Asli in Malaysia, and this study also determined factors contributing to the diet quality of this particular population.

## 5. Conclusions

In general, the diet quality of the *Orang Asli* women was poor, with low compliance to the Malaysian dietary recommended intake of vegetables, fruits, meat, poultry and eggs, legumes, and milk. Factors contributing to poor diet were women being single, divorced, or widowed, living in poor households, experienced food insecurity and having increased intake of fat. However, nutrition knowledge was independently related to diet quality. The findings from this study warrant the efforts to promote improved diet quality among lower income households in order to prevent diet-related NCDs. Additionally, nutrition promotion campaigns and intervention endeavors focusing on enhancing nutrition knowledge should be continued to improve diet quality and health status among this population.

## Figures and Tables

**Table 1:** Criteria scoring for Malaysian Healthy Eating Index components.

Components	Score Range	Criteria for Maximum Score 0	Criteria for Score 8	Criteria for Maximum Score 10
Food groups				
Grains and cereals	0–10	0		4–8 servings ^1^
Vegetables	0–10	0		3 servings ^1^
Fruits	0–10	0		2 servings ^1^
Meat, poultry and eggs	0–10	0		½–2 servings ^1^
Fish and seafoods	0–10	0		1 servings ^1^
Legumes	0–10	0		½–1 servings ^1^
Milk and dairy products	0–10	0		1–3 servings ^1^
Nutrients				
Total fat	0–10	≥35% energy ^2^ from fat		≤30% energy ^1^ from fat
Sodium	0–10	≥4200 mg ^2^	2400 mg ^1^	≤2000 mg ^1^

^1^ Based on the Malaysian Dietary Guidelines 2010. ^2^ Based on the Malaysian Adult Nutrition Survey 2003.

**Table 2:** Malaysian Healthy Eating Index (HEI) scoring for nutrients.

HEI Score	0	1	2	3	4	5	6	7	8	9	10
Energy intake from fat (%)	≥35.0	34.5	34.0	33.5	33.0	32.5	32.0	31.5	31.0	30.5	≤30.0
Sodium (mg)	≥4200	3975	3750	3525	3300	3075	2850	2625	2400	2200	≤2000

**Table 3:** Socioeconomic characteristics, nutrition knowledge, and food security status of the respondents (*n* = 222).

Variables	*n* (%)	Mean ± SD
Age (years)		36.5 ± 11.5
19–29	73 (32.9)	
30–50	111 (50.0)	
51–59	38 (17.1)	
Marital status		
Single/Divorced/Widowed	42 (18.9)	
Married	180 (81.1)	
Education (years)		6.1 ± 3.7
No school	35 (15.8)	
Primary school	110 (49.5)	
Secondary school	68 (30.6)	
College/University	9 (4.1)	
Employment status		
Working	84 (37.8)	
Housewife	138 (62.2)	
Household income ^1^ (USD)		228.2 ± 215.9.
≤140.1	84 (37.8)	
140.2–224.6	58 (26.1)	
>224.6	80 (36.1)	
Nutrition knowledge (%)		33.0 ± 19.6
Poor	184 (82.9)	
Moderate	31 (14.0)	
Good	7 (3.1)	
Food security status		
Food secure	38 (17.1)	
Household food insecure	65 (29.3)	
Individual food insecure	52 (23.4)	
Child hunger	67 (30.2)	

Note: *n*, number; SD, standard deviation. United States Dollar (USD) = 4.14 Malaysian Ringgit (MYR). ^1^ Brief Household Income & Poverty Statistics Newsletter by Economic Planning Unit 2014 [28].

**Table 4:** Energy and nutrients intake of respondents by age groups (*n* = 222).

Nutrients	19–29 (*n* = 73)			30–50 (*n* = 111)			51–59 (*n* = 38)		
	Mean ± SD	RNI	%RNI	Mean ± SD	RNI	%RNI	Mean ± SD	RNI	%RNI
Energy (kcal)	1370 ± 303	1610	85.0	1303 ± 283	1660	78.5	1324 ± 218	1660	79.8
Carbohydrate (g)	185.5 ± 43.3	-		174.8 ± 41.3	-		181.6 ± 35.8	-	
Protein (g)	56.7 ± 17.5	53	107.0	55.5 ± 16.9	52	106.7	56.0 ± 12.3	52	107.7
Fat (g)	45.6 ± 14.1	-		41.7 ± 12.7	-		41.9 ± 10.0	-	
Calcium (mg)	417.5 ± 193.7	1000	41.8	389.8 ± 152.0	1000	39.0	489.0 ± 233.1	1200	40.8
Iron (mg)	9.8 ± 4.4	29	33.8	9.8 ± 4.1	29	33.8	11.0 ± 4.3	11	100.0
Zinc (mg)	2.4 ± 1.5	4.7	51.1	2.4 ± 1.7	4.6	52.2	2.7 ± 1.7	4.6	58.7
Selenium (µg)	15.4 ± 15.0	25	61.6	13.5 ± 14.4	24	56.3	13.6 ± 13.1	24	56.7
Vitamin A (µg)	542.6 ± 341.9	600	90.4	531.2 ± 319.3	600	88.5	647.0 ± 321.7	600	107.8
Thiamin (mg)	0.6 ± 0.3	1.1	54.5	0.6 ± 0.2	1.1	54.5	0.6 ± 0.2	1.1	54.5
Riboflavin (mg)	0.8 ± 0.3	1.1	72.7	0.8 ± 0.3	1.1	72.7	0.8 ± 0.3	1.1	72.7
Niacin (mg)	7.9 ± 3.3	1.4	56.4	7.7 ± 3.1	1.4	55.0	7.8 ± 2.6	1.4	55.7
Vitamin C (mg)	50.3 ± 41.8	70	71.9	54.5 ± 36.8	70	77.9	75.0 ± 41.2	70	107.1

SD, standard deviation; RNI, recommended nutrient intake.

**Table 5:** Household income and nutrition knowledge correlated with component scores of Malaysian Healthy Eating Index (HEI).

Variables	Serving Size/Day Mean ± SD	Recommended Serving Size/Day ^1^	Mean Score ± SD/Median (IQR) Score of Malaysian HEI	Household Income		Nutrition Knowledge	
				r/r_s_	*p* Value	r/r_s_	*p* Value
Malaysian HEI ^a^ (%)			45.3 ± 7.5	0.242	0.000 ***	0.150	0.026 *
Grains and cereals ^a^	4.7 ± 1.1	4–8	8.4 ± 1.6	0.163	0.015 *	0.015	0.820
Vegetables ^a^	1.1 ± 0.8	3	3.6 ± 2.2	0.155	0.021*	0.171	0.027 *
Fruits ^b^	0.0 (0.0)	2	0.0 (0.0)	0.264	0.000 ***	0.250	0.000 ***
Meat, poultry and eggs ^a^	0.6 ± 0.6	0.5–2	4.3 ± 3.5	0.180	0.007 **	0.106	0.115
Fish and seafoods ^a^	1.6 ± 0.9	1	8.5 ± 2.4	0.040	0.554	−0.047	0.485
Legumes ^b^	0.0 (0.0)	0.5–1	0.0 (0.0)	0.223	0.001 **	0.069	0.305
Milk and dairy products ^b^	0.0 (0.0)	1–3	0.0 (0.0)	0.127	0.058	0.020	0.772
Total fat^a^ (% from total energy intake)	29.0 ± 5.6	-	7.3 ± 4.0	−0.109	0.104	−0.042	0.533
Sodium ^a^ (mg)	2441.0 ± 779.5	-	7.4 ± 2.7	−0.094	0.161	0.076	0.261

SD, standard deviation; IQR, interquartile range. ^1^ Serving size based on recommendations of the Malaysian Dietary Guidelines (NCCFN, 2010). ^a^ Pearson correlation with reported r; ^b^ Spearman rank correlation with reported r_s_. * Significant at *p* < 0.05; ** Significant at *p* < 0.01; *** Significant at *p* < 0.001.

**Table 6:** Predictors of diet quality from the multiple linear regression model.

Variables	Unstandardized B	95% Confidence Interval for B	β Coefficients	*p* Value
Married	3.450	1.503–5.398	0.181	0.001 **
Household income (RM)	5.457	2.787–8.128	0.237	0.000 ***
Nutrition knowledge	0.136	−0.081–0.353	0.071	0.218
Food insecurity	−2.979	−5.046–−0.912	−0.151	0.005 **
Adjusted carbohydrate (% kcal)	0.087	−0.101–0.274	0.081	0.364
Adjusted fat (% kcal)	−0.583	−0.813–−0.353	−0.438	0.000 ***

*R*^2^ = 0.416. *F* (6, 215) = 23.460, *p* < 0.001; controlled for dietary misreporting. ** Significant at *p* < 0.01; *** Significant at *p* < 0.001.

## Supplementary Materials

The following are available online at http://www.mdpi.com/2072-6643/11/1/135/s1. Table S1: Serving size for each food group. Table S2: Recommended serving sizes according to food groups based on total energy intake. Table S3: Relationship between socio-demographic characteristics, nutrition knowledge, food security status, adjusted macronutrients, and diet quality from the simple linear regression model.
