# Supplementary material for: Predictors of Diet Quality as Measured by Malaysian Healthy Eating Index among Aboriginal Women (Mah Meri) in Malaysia
Source: Nutrients. 2019 Jan 10;11(1):135. doi: 10.3390/nu11010135 (PMC6356371; doi:10.3390/nu11010135)
Supplement: Supplementary file 1 [file nutrients-11-00135-s001.pdf]

Supplementary Table S1. Serving size for each food group [17,22,23]

Food groups	Serving size
Cereals, cereal products and tubers (30g carbohydrate per serving)	- 2 slices of white bread/ wholemeal bread<br>- 6 pieces of cream cracker biscuits<br>- 1 cup of wet noodles/ *kuey teow*<br>- 1½ cup of *bihun* (soaked)<br>- 2 whole potatoes<br>- 2 pieces of *putu mayam*<br>- 1 cup/ 2 scoops of cooked white rice<br>- 2 cups of plain porridge<br>- 1 cup of sweet potato/ *yam*/ tapioca
Vegetables	- ½ cup of cooked green leafy vegetables with edible stem<br>- ½ cup of cooked fruit vegetables<br>- 1 cup of raw *ulam*
Fruits (15g carbohydrate per serving)	- 1 whole apple/ chinese pear/ mango/ *ciku*<br>- 1 whole medium size banana *berangan*/ mandarin orange<br>- 2 whole banana *emas*<br>- ½ whole guava/ pear<br>- 1 slice of papaya/ pineapple/ watermelon<br>- 3 *ulas* durian<br>- 8 small grapes<br>- 4 small prunes<br>- 1 dessert spoon raisins
Meat, poultry and eggs (14g protein per serving)	- 2 pieces of lean beef (7.5 cm x 9 cm x 0.5 cm)<br>- 1 piece of chicken drumstick<br>- 2 whole chicken eggs<br>- 12 whole quail eggs<br>- 2 pieces of chicken liver
Fish and seafoods (14g protein per serving)	- 2/3 cups of anchovies (head removed)<br>- 1 medium *ikan kembung*/ *ikan selar*<br>- 1 piece of *ikan tenggiri* (14 cm x 8 cm x 1 cm)<br>- 1 cup of cockles without shells<br>- 2 medium squid
Legumes (7g protein per serving)	- 1 cup of chickpea/ dhal<br>- 1½ cups of green/ mung bean/ canned baked bean<br>- 2 pieces of *tempe* (12 cm x 9 cm x 0.5 cm)/ *taukua* (5.5 cm x 6 cm x 2.5 cm)/ *tauhu* (6 cm x 7.5 cm x 3.5 cm)<br>- 1½ glass of unsweetened soy bean milk
Milk and dairy products (7g protein per serving)	- 1 slice of cheese (8.4 cm x 8.4 cm x 0.3 cm)<br>- 1 glass of low fat milk<br>- 2/3 cup of evaporated milk<br>- 4 dessert spoons of powdered milk<br>- 1 cup of yoghurt

Supplementary Table S2. Recommended serving sizes according to food groups based on total energy intake [22]

**Food groups**	**Sedentary women 1500 cal/day**	**Moderate active women 2000 cal/day**	**Very active women 2500 cal/day**
Grains and cereals[a]	4 servings	6 servings	8 servings
Vegetables	3 servings	3 servings	3 servings
Fruits[b]	2 servings	2 servings	2 servings
Meat, poultry and eggs[c]	½ serving	1serving	2servings
Fish and seafoods[c]	1 serving	1 serving	1 serving
Legumes[d]	½ serving	1 serving	1 serving
Milk and dairy products[d]	1 serving	2 servings	3 servings

[a]Based on 30g carbohydrate per serving
[b]Based on 15g carbohydrate per serving
[c]Based on 14g protein per serving
[d]Based on 7g protein per serving

Supplementary Table S3. Relationship between socio-demographic characteristics, nutrition knowledge, food security status, adjusted macronutrients and diet quality from the simple linear regression model

**Variables**	**Unstandardized B**	**95 % Confidence Interval for B**	**p value**
Age (years)	0.046	-0.040 – 0.132	0.296
Married	3.555	1.072 – 6.038	0.005**
Education (years)	0.290	0.023 – 0.558	0.034*
Employed	-0.194	-2.236 – 1.847	0.851
Household income (RM)	0.002	0.001 – 0.003	0.000***
Nutrition knowledge	0.285	0.035 – 0.535	0.026*
Food insecurity	-3.105	-5.701 - -0.509	0.019*
Adjusted carbohydrate (% kcal)	0.383	0.250 – 0.515	0.000***
Adjusted protein (% kcal)	-0.233	-0.561 – 0.096	0.164
Adjusted fat (% kcal)	-0.586	-0.745 – -0.427	0.000***

*Significant at p<0.05; **Significant at p<0.01; ***Significant at p<0.001
